# Morphology of the murine choroid plexus: Attachment regions and spatial relation to the subarachnoid space

**DOI:** 10.3389/fnana.2022.1046017

**Published:** 2022-10-31

**Authors:** Theresa Greiner, Katerina Manzhula, Louise Baumann, Hannes Kaddatz, Jens Runge, Jonas Keiler, Markus Kipp, Sarah Joost

**Affiliations:** ^1^Institute of Anatomy, Rostock University Medical Center, Rostock, Germany; ^2^Department of Ophthalmology, Rostock University Medical Center, Rostock, Germany

**Keywords:** murine choroid plexus, ventricle, subarachnoid space, attachment, micro-computed tomography, mus musculus, x-ray micro-tomography, immune cell migration

## Abstract

The choroid plexus has recently been identified as a possible migration route for peripheral immune cells into the central nervous system. For future investigation of this route, profound knowledge of the morphology of the murine choroid plexus is a prerequisite. We here present a detailed morphological description of the murine choroid plexus, its attachment regions as well as its spatial relation to the subarachnoid space. We used micro-computed tomography of immersion-contrasted fixated brains to generate three-dimensional models of the ventricle system and the choroid plexus and aligned micro-computed tomography-based sections with histological paraffin-embedded sections after immunohistochemical labeling of the basal lamina and choroid plexus epithelium marker proteins (laminin and aquaporin 1). The murine choroid plexus is located in all four ventricles and is attached to the brain parenchyma in narrow attachment regions with a specific morphology in each ventricle. While in the lateral and fourth ventricle, the attachment site is formed by thin tissue bridges, the choroid plexus attachment in the third ventricle has a more complex V-like shape. In all ventricles, the choroid plexus is in close spatial relationship with the subarachnoid space that extends from the brain surface along physiologic openings toward the choroid plexus. In summary, we here provide a description of the morphology of the murine ventricle system and choroid plexus, the attachment regions of the choroid plexus and its connection to the subarachnoid space, as well as a three-dimensional model of the ventricles, the choroid plexus, and the subarachnoid space to facilitate a spatial understanding of these complex structures.

## Introduction

The central nervous system (CNS) encloses four cavities filled with cerebrospinal fluid (CSF), the ventricles. The paired lateral ventricles are connected *via* interventricular foramina to the third ventricle, which is connected to the fourth ventricle *via* the cerebral aqueduct. The fourth ventricle is continuous to the CSF-filled subarachnoid space that surrounds the outside of the CNS.

In all four ventricles, CSF is produced by the choroid plexus (CP), a ruffled structure consisting of the CP epithelium surrounding the strongly vascularized CP stroma. The CP epithelium is the site of CSF production and forms the blood-CSF-barrier (Ghersi-Egea et al., 2018). Furthermore, the CP has recently been identified as a site of immune cell surveillance of the CNS and might play a critical role as an entry route for peripheral immune cells into the CNS under physiological and pathological conditions (Engelhardt et al., 2001; Schwartz and Baruch, 2014; Strominger et al., 2018).

Classically, the route of immune cell recruitment into the CNS has been considered to involve breaching and crossing the blood-brain-barrier at the level of parenchymal post-capillary venules. However, elevated numbers of peripheral immune cells in the CSF are a common observation in various inflammatory neurologic conditions. On the one hand, they are explained by breaching of these cells through the blood-CSF-barrier at the CP epithelium after egressing from CP stroma vessels (Kivisäkk et al., 2003; Reboldi et al., 2009). On the other hand, it has been shown that peripheral immune cells can also migrate through the walls of meningeal vessels into the CSF-filled subarachnoid space (Schläger et al., 2016). From the CSF, immune cells might reach the CNS parenchyma either by crossing the ependymal cell layer lining all ventricles or the pia mater covering the outer surface of the CNS. Additionally, it has been proposed that immune cell migration into the depth of the CNS parenchyma from the subarachnoid space can occur *via* the perivascular space of penetrating vessels (Greiner and Kipp, 2021). A further possible route has been raised by Llovera and colleagues, who propose that immune cells may migrate directly from the CP stroma into the CNS parenchyma via the attachment region of the CP (Llovera et al., 2017).

The prerequisite for analyzing the anatomical routes of immune cell recruitment into the CNS is a profound knowledge of the three-dimensional anatomy of the ventricle system and the incorporated CP. While detailed references are available for the anatomy of the human ventricle and CP, the ventricles and especially the CP of the mouse, the commonly used model organism for basic neurologic research, have not been morphologically described in considerable detail. Especially three-dimensional visualization of the entire murine ventricle system but not the CP has only been reconstructed from histological data, leading to artifact-prone results with limited resolution (Taguchi and Chida, 2003) and from micro-computed tomography (micro-CT, Chiani et al., 2019). Of note, a recent study demonstrated a three-dimensional reconstruction of the fourth venticle’s CP from clarified rat tissue with a detailed focus on choroidal blood vessels (Perin et al., 2021).

Here, we describe the morphology of the murine ventricular system and the CP by means of micro-CT and three-dimensional reconstruction to promote spatial understanding of this complex structure. With regard to possible immune cell migration routes, special emphasis is put on the localization of the attachment regions of the CP toward the CNS parenchyma and the spatial relationship of the subarachnoid space to the inner ventricular system.

## Materials and methods

### Animals and transcardial perfusion

Adult C57BL/6 mice (*n* = 9 for histological and *n* = 4 for micro-CT studies) were obtained from the stockbreeding of the central animal facility of Rostock University Medical Center. The mice were maintained at a maximum of five animals per cage with food and water *ad libitum*. The mice were kept under standard conditions (12°h light/dark cycle, controlled temperature 22°C ± 2°C). Cages were changed once per week. All experimental procedures concerning animals were performed according to the Federation of European Laboratory Animal Science Associations recommendations, in accordance with the German Animal Welfare Law, and approved by the local authorities.

Mice were anesthetized with an overdose of ketamine (750 mg kg^–1^ i.p.) and xylazine (50 mg kg^–1^ i.p.) and transcardially perfused with 20 ml of ice-cold phosphate-buffered saline (PBS) followed by 50 ml of neutral buffered 3.7% formaldehyde solution (pH 7.4). Brains were dissected and post-fixated in buffered formaldehyde solution overnight.

### Micro-computed tomography and three-dimensional reconstruction

Fixated murine brains were washed in PBS for two days. Samples were transferred into approx. 75 ml of a contrast agent composed of 1% aqueous Lugol’s iodine (iodine-potassium iodide) for at least 18 days on a shaking table (60 rpm). The contrast agent was exchanged after 14 days. Prior to micro-CT, the samples were washed in PBS for 0.5-2 h to remove residues of the contrasting agent. In preparation for micro-CT, two samples were mounted in microcentrifuge tubes filled with PBS. The position of the sample was fixed with a stiff plug of wet cellulose tissue gently pressed on its top. The other two samples were embedded in 0.5% agarose to avoid movements during the scans. Virtual image stacks were obtained by micro-CT using a Xradia Versa 410 x-ray microscope (Carl Zeiss Microscopy GmbH, Jena, Germany). Micro-CT scans were conducted at 60 kV and 133 μA using the software Scout and Scan v.11.1 (Carl Zeiss Microscopy GmbH). Overview scans of the whole brains were performed with the 0.4x objective (voxel size: 12.8-15.4 μm, sample rotation: 220°–360°, projection increment 0.22°–0.29°). Detailed scans of the CP (in the lateral, third and fourth ventricle) were performed with the 4x objective (voxel size: 2.2–4.3 μm, sample rotation: 196°–360°, projection increment: 0.11°–0.20°).

**FIGURE 1:**
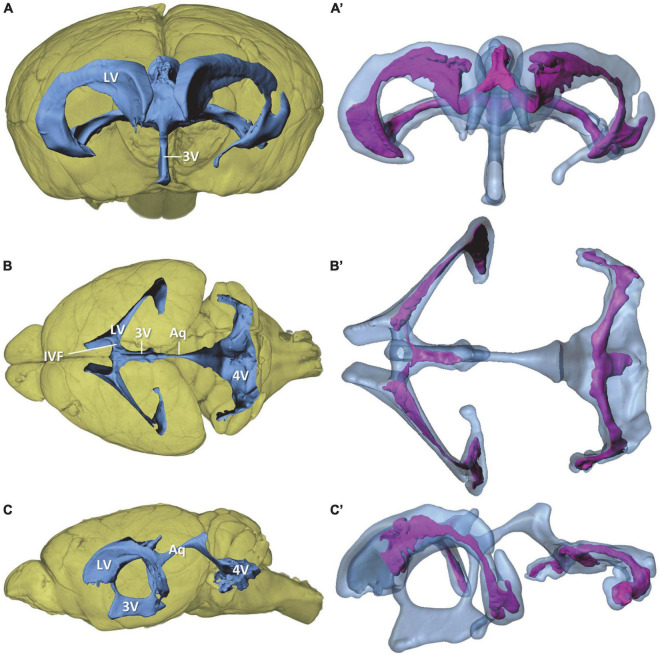
Overview of the murine ventricular system and the choroid plexus within the ventricles. Volume rendering of the mouse brain (yellow) with surface models of the ventricular system (blue) and choroid plexus (purple) based on micro-computed tomography scans of a mouse brain. **(A,A’)** View from rostral; **(B**,**B’)** view from dorsal, **(C,C’)** view from lateral. 3V, third ventricle; 4V, fourth ventricle; Aq, aqueduct; IVF, interventricular foramen; LV, lateral ventricle.

Analysis of micro-CT data and the visualization was conducted with the software Imaris (version 8.4, Bitplane, Switzerland). Firstly, the ventricular system and the CP were manually segmented roughly to exclude neighboring structures. These segmentations were used to mask (extract) the respective volumetric information and create separate channels. Secondly, in order to generate detailed surface reconstructions, an automated, threshold-based segmentation/extraction was applied to the (first) masked channels by adjusting the contrast values. For the ventricular system, the gray values of the masked channel have been inverted before the automated segmentation. The resultant detailed reconstructions were used for final masking and to prepare the images and three-dimensional surface models (embedded in Figures 1, 3–6, the three-dimensional model is provided in Supplementary material 1).

**FIGURE 2:**
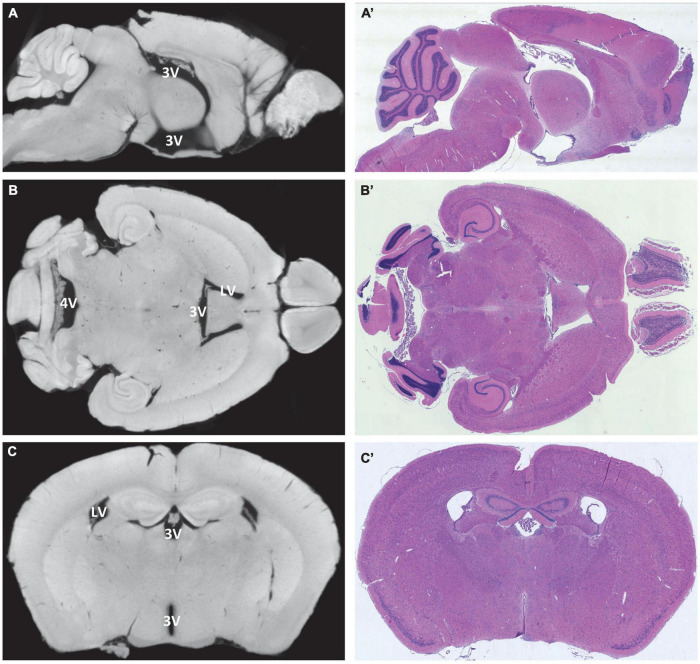
Alignment of virtual and histological sections. On the left, virtual sections from micro-computed tomography scans, and on the right, hematoxylin-eosin-stained paraffin-embedded sections from the same region and sectional plane. **(A,A’)** Median section in sagittal orientation, the choroid plexus is visible in the third ventricle and fourth ventricle, lateral ventricles are not visible; **(B**,**B’)** horizontal section at the level of the interventricular foramen between the lateral ventricles and the third ventricle, the choroid plexus is visible in all four ventricles; **(C**,**C’)** coronal section at the level of the rostral hippocampus with lateral ventricles and third ventricle, the choroid plexus is visible in both ventricles. LV, lateral ventricle; 3V, third ventricle; 4V, fourth ventricle.

**FIGURE 3:**
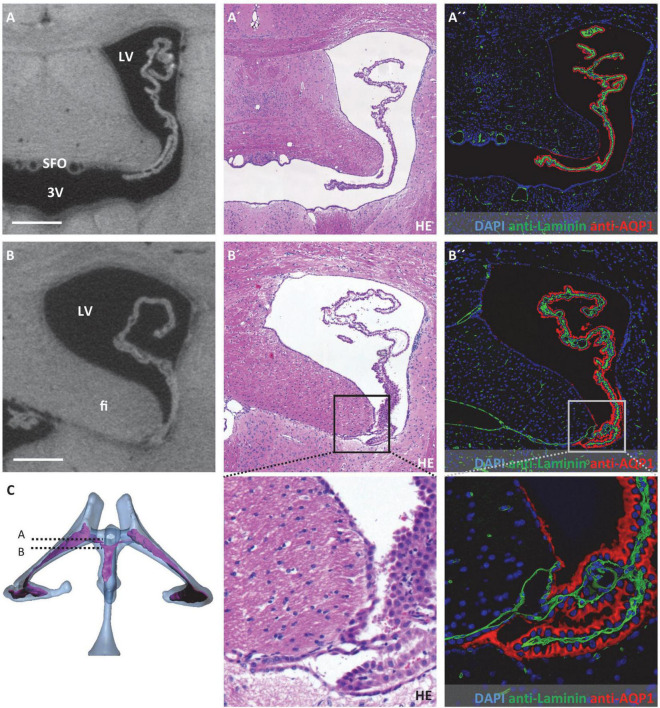
Attachment regions of the choroid plexus in the lateral ventricle. On the left, virtual sections from micro-computed tomography scans; in the middle, hematoxylin-eosin staining; and on the right, immunofluorescence double labeling with anti-laminin (green), anti-aquaporin1 (AQP1, red), and nuclear staining with 4′,6-diamidino-2-phenylindole (DAPI, blue). **(A–A”)** Coronal section at the level of the interventricular foramen that connects the lateral ventricle and third ventricle, the subfornical organ is located at the roof of the third ventricle, the choroid plexus is neither attached at the level of the interventricular foramen nor rostral to it; **(B–B”)** attachment of the choroid plexus occipital to the interventricular foramen, the epithelium of the choroid plexus transitions into the ependyma at the tip of the fimbriae hippocampi, the basal laminae pass along between the fimbriae hippocampi and diencephalon as seen in **(B”)**, below **(B’,B”)** enlargements of the attachment site; **(C)** illustration of sectional planes based on three-dimensional reconstruction of ventricular system with choroid plexus. Scale bars in **(A,B)** 200 μm. LV, lateral ventricle; 3V, third ventricle; fi, hippocampal fimbria; SFO, subfornical organ.

**FIGURE 4:**
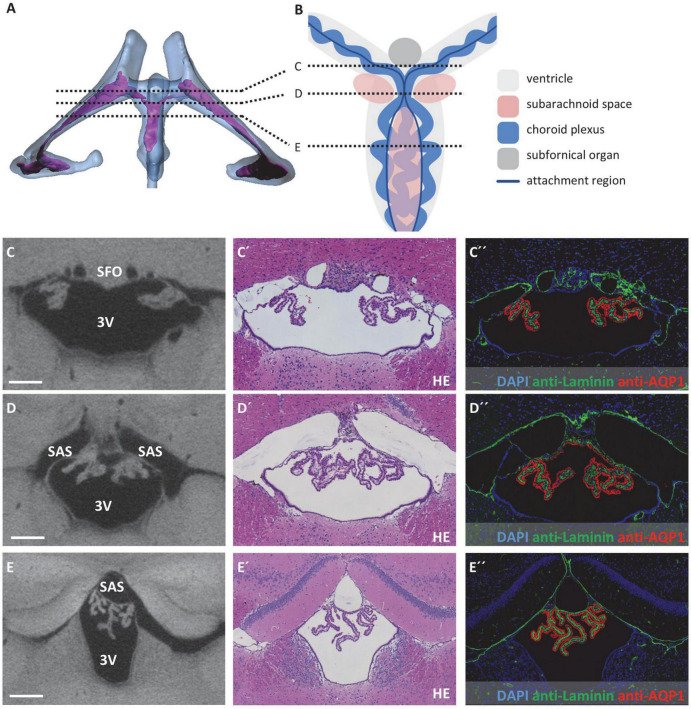
Attachment regions of the choroid plexus in the third ventricle. **(A)** Illustration of sectional planes in three-dimensional reconstruction of the lateral and third ventricle (blue) and the choroid plexus (purple) in a dorsal view. **(B)** Schematic drawing of the third ventricle in dorsal view with choroid plexus, subfornical organ, subarachnoid space, and sectional planes of **(C–E)**. Created with BioRender.com **(C–E)** On the left, sectional planes from micro-computed tomography scan; in the middle, hematoxylin-eosin staining; and on the right, immunofluorescence double labeling with anti-laminin (green), anti-aquaporin1 (AQP1, red), and nuclear staining with 4′,6-diamidino-2-phenylindole (DAPI, blue). **(C–C”)** Coronal section at the level of the subfornical organ occipital to the interventricular foramen; **(D–D”)** coronal section occipital to the subfornical organ with median attachment site of the choroid plexus and lateral cavities of subarachnoid space; **(E–E”)** coronal section at the level of the rostral hippocampus, the choroid plexus is attached to the roof of the third ventricle via two lateral attachments, the space between the two attachment sites belongs to the subarachnoid space. Scale bars in **(C–E)** 200 μm. 3V, third ventricle; SAS, subarachnoid space; SFO, subfornical organ.

**FIGURE 5:**
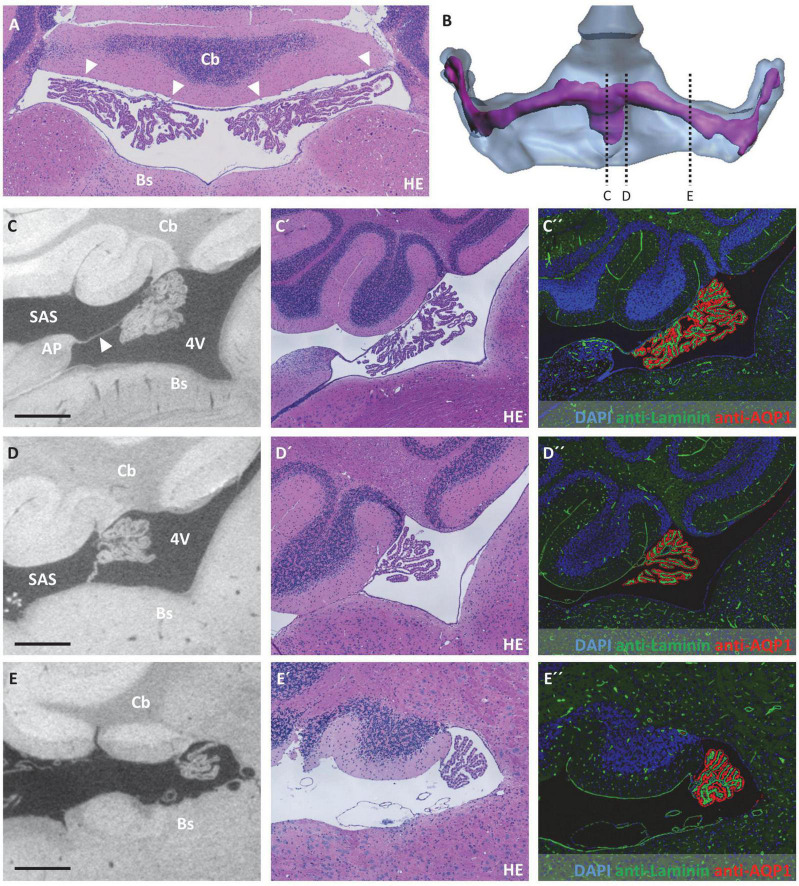
Attachment regions of the choroid plexus in the fourth ventricle and opening of the fourth ventricle to the subarachnoid space. **(A)** Horizontal section of the fourth ventricle after hematoxylin-eosin staining, the broad attachment zone of the choroid plexus toward the cerebellum is marked by arrowheads. **(B)** Illustration of sectional planes in three-dimensional reconstruction of the fourth ventricle (blue) and the choroid plexus (purple) in a dorsal view. **(C–E)** On the left, virtual sections from micro-computed tomography scan; in the middle, hematoxylin-eosin staining; and on the right, immunofluorescence double labeling with anti-laminin (green), anti-aquaporin1 (AQP1, red), and nuclear staining with 4′,6-diamidino-2-phenylindole (DAPI, blue). **(C–C”)** Fourth ventricle in median sectional plane in sagittal orientation, the choroid plexus is attached to the cerebellum and connected to the area postrema of the brainstem by a delicate tissue bridge (arrowhead); **(D–D”)** fourth ventricle in paramedian sectional plane in sagittal orientation, attachment of choroid plexus to cerebellum and brainstem; **(E–E”)** fourth ventricle in sagittal sectional plane, attachment of choroid plexus to the cerebellum and opening of the fourth ventricle toward the subarachnoid space. Scale bars in **(C–E)** 200 μm. 4V, fourth ventricle; AP, area postrema; Bs, brain stem; Cb, cerebellum; SAS, subarachnoid space.

**FIGURE 6:**
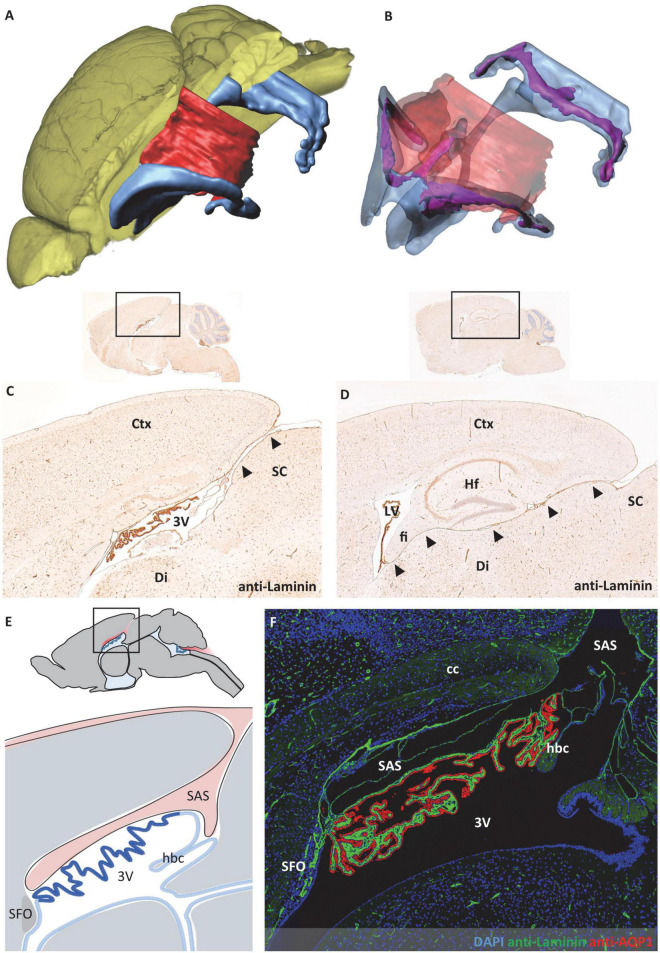
Relationship between subarachnoid space and third ventricle. The subarachnoid space extends from occipitally beneath the cortex and rostrally above the third ventricle. **(A)** Volume rendering of the brain (yellow) with surface models of the ventricular system (blue) and subarachnoid space (red) to illustrate their spatial relation. **(B)** Transparent representation of the subarachnoid space and the ventricular system show the choroid plexus (purple) in the ventricular lumen. **(C,D)** Sagittal sections with immunohistochemical labeling with anti-laminin and faint hemalum counterstaining depict the connection of the subarachnoid space and the roof of the third ventricle in the mediosagittal plane in **(C)** (arrowheads) and the connection of the subarachnoid space and the lateral ventricle in a lateral sectional plane in **(D)** (arrowheads). **(E)** Schematic of the median region in sagittal orientation, the subarachnoid space (red) is in contact with the ventricular system above the third ventricle. Created with BioRender.com. **(F)** Immunofluorescence double labeling with anti-laminin (green), anti-aquaporin1 (AQP1, red), and nuclear staining with 4′,6-diamidino-2-phenylindole (DAPI, blue) of the same region as shown in **(E)**. 3V third ventricle, cc corpus callosum, Ctx, cortex; Di, diencephalon; fi, hippocampal fimbria; hbc, habenular commissure; Hf, hippocampal formation; LV, lateral ventricle; SC, superior colliculus; SAS, subarachnoid space; SFO, subfornical organ.

### Histology and immunofluorescence labeling

All histological studies and immunofluorescence labeling were performed using paraffin-embedded 5 μm-thick brain sections in coronal, sagittal, and horizontal orientations. Hematoxylin-eosin (HE) staining and anti-aquaporin1 (AQP1)/anti-laminin immunofluorescence double staining were performed on selected consecutive sections. For HE staining, sections were deparaffinized and rehydrated. Sections were incubated in Mayer’s hemalum solution for 10 min. After dipping in hydrochloric acid ethanol, sections were washed in running tap water. The sections were then washed in distilled water and incubated in eosin solution. The staining intensity of eosin was differentiated in 70% ethanol. After dehydration, the sections were mounted with Depex^®^.

For immunofluorescence labeling, sections were deparaffinized and rehydrated. Antigens were unmasked by microwave heating to the boiling point in tris(hydroxymethyl)aminomethane/ethylenediamine tetraacetic acid (Tris/EDTA) buffer (pH 9.0) for 10 min. The sections were washed in PBS and then incubated for 1 h with 5% normal donkey serum in PBS. After draining the blocking serum, the sections were incubated overnight at 4°C with the primary antibodies diluted in blocking solution (anti-AQP1, Abcam Cat# ab9566, RRID:AB_296494, 1:600; anti-Laminin, Abcam Cat# ab11575, RRID:AB_298179, 1:300). Appropriate negative controls (omission of primary antibodies and cross controls of primary antibody and mismatched secondary antibody) were performed in parallel. After washing in PBS, the sections were incubated with the appropriate secondary antibodies diluted 1:250 in blocking serum (Alexa Fluor™ 594 donkey anti-mouse, Molecular Probes Cat# A-21203, RRID:AB_141633; Alexa Fluor™ 488 donkey anti-rabbit, Abcam Cat# ab150065, RRID:AB_2860569) for 2 h at room temperature. The sections were then washed in PBS and mounted in 4′,6-diamidino-2-phenylindole (DAPI)-containing mounting medium (Fluoroshield™ with DAPI) for the staining of cell nuclei. Stained and processed sections were documented using a brightfield/epifluorescence microscope (Leica DM6 B) and the software Leica Application Suite X (version 3.7.0.20979, 2019, Germany).

For immunohistochemistry, sections were deparaffinized, rehydrated, and antigens were unmasked by heating in Tris/EDTA buffer. After washing in PBS, the sections were incubated for 1 h in 5% normal goat serum in PBS. After draining the blocking serum, the sections were incubated overnight at 4°C with the primary antibody diluted in blocking solution (anti-Laminin, Abcam Cat# ab11575, RRID:AB_298179, 1:500). Appropriate negative controls (omission of primary antibody) were performed in parallel. The slides were treated with 0.35% hydrogen peroxide (H_2_O_2_) the next day in PBS for 30 min. After washing in PBS, the slides were incubated in biotinylated secondary antibody (goat anti-rabbit IgG, Vector Laboratories Cat# BA-1000, RRID:AB_2313606, 1:200) for 1 h at ambient temperature and then in peroxidase-coupled avidin-biotin complexes for 1 h (ABC kit; Vector Laboratories, Peterborough, UK). The antigenic sites were detected by a reaction with 3,3’-diaminobenzidine (DAKO, Hamburg, Germany) and H_2_O_2_ yielding a brownish deposit. Stained and processed sections were documented using a brightfield/epifluorescence microscope and the software Leica Application Suite X.

## Results

For the morphological analysis of the murine ventricular system and CP, we performed micro-CT of fixated and immersion-contrasted adult murine brains and reconstructed the total brain volume, the four ventricles, and the CP within the ventricles (Figures 1A–C and Supplementary material 1). The lateral ventricles have the shape of a tilted flattened “C” that is broader at the anteromedial and posterolateral portion than in the central section. The anteromedial portion narrows into the interventricular foramen that forms the connection to the third ventricle by forming a dorsally ascending course toward the median plane. The third ventricle is a narrow ring in the median plane with a mean lateral width of 130 μm. The basal and parietal aspects of the ring are enlarged in height and width, respectively. The cerebral aqueduct connects the parietal portion of the third ventricle with the fourth ventricle. The fourth ventricle possesses a complex shape that can be divided into an upper and a lower portion. The upper portion is connected to the slightly ascending aqueduct, and, from there, it forms a downward bend with a dorsally oriented extension. While descending toward the lower part of the fourth ventricle, the ventricular lumen broadens into an irregular-shaped, almost horizontal plane that comprises the lower part of the ventricle. This part of the fourth ventricle has lateral expansions, almost reaching the width of the lateral ventricles. The most lateral portions of the ventricle are oriented toward anterior.

The CP is located in all four ventricles but does not fill all cavities of the ventricular system (Figures 1A’–C’). It spans almost the entire lateral ventricle, except for the most anterior portion. The largest CP expansions can be found in the posterolateral section and in the anteromedial section of the lateral ventricles close to the interventricular foramen. The CPs from both lateral ventricles proceed through the interventricular foramina, merge at the roof of the third ventricle and extend posteriorly within the third ventricle. The lower portion of the third ventricle, the mesencephalic aqueduct, and the upper portion of the fourth ventricle do not contain CP. In the lower portion of the fourth ventricle, a large mass of CP extends over the whole width into the most lateral sections of the ventricle. In the median plane, the CP additionally extends posteriorly.

For a more detailed analysis of the morphological features, paraffin-embedded adult murine brains were sectioned in frontal, horizontal, and sagittal cutting planes and aligned to the micro-CT data (Figure 2). The morphology of micro-CT-based (virtual) and histologic sections resulted to be highly congruent. Occasionally, however, morphological spaces and fissures opened to varying degrees in the two visualization methods. From our data, we cannot conclude if these are interindividual differences or shrinkage artifacts resulting from the sample preparation procedure (Supplementary Figure 1).

Concerning possible migration routes of peripheral immune cells into the CNS, we analyzed the attachment regions of the CP toward the brain parenchyma. For detection and description of these attachment regions, we used a combination of micro-CT data and histological sections after HE staining and immunohistochemical labeling of the water channel protein AQP1 expressed by CP epithelium and the basal lamina component laminin. Of note, the ependymal cell layer that lines all ventricular spaces does not have a basal lamina. Laminin-labeling was therefore used to distinguish brain parenchyma facing ventricular cavities (basal lamina absent) and brain parenchyma facing the CP or the subarachnoid space (basal lamina present).

In the lateral ventricles, the CP spans almost the whole ventricular lumen. There is no attachment of the CP toward the CNS parenchyma anteriorly to the interventricular foramen (Figure 3A). Posterior to the interventricular foramen, the CP is attached to the CNS parenchyma in a narrow zone alongside the tip of the hippocampal fimbria and the opposing portion of the diencephalon (Figure 3B). This attachment presents as delicate tissue bridges with a continuous basal lamina (Figure 3B”) between which extensions of the subarachnoid space reach the CP stroma (please see below for connection of the lateral ventricle and subarachnoid space). In histological sections with artificial dilation of the gap between hippocampal fimbria and diencephalon, these tissue bridges are easier to demarcate and present as clearly separated from each other (Supplementary Figure 2).

At the roof of the third ventricle, attachment of the CP toward the brain parenchyma proved to be more complex and is therefore schematically depicted in Figures 4A,B. In the most anterior part of the third ventricle roof, two strands of CP protrude from the lateral ventricles by passing the interventricular foramen, resulting in two separate attachment areas at the third ventricle’s roof divided by the subfornical organ (Figure 4C). As the two strands of CP combine, they form one medial attachment point posterior to the subfornical organ (Figure 4D). Bilateral to this attachment point, the subarachnoid space forms two cavities separated from the third ventricle by a thin tissue bridge (please see below for the connection of the third ventricle and subarachnoid space). Posterior to this medial CP attachment point, an extension of the subarachnoid space is localized in the midline of the third ventricle’s roof (Figure 4E). Therefore, the attachment regions of the CP again divide into two strands that continue directly lateral to the extension of the subarachnoid space (dark blue lines in Figure 4B between sections D and E). However, the stroma and epithelium of both sides of the CP remain connected and form a bulk of CP tissue beneath the subarachnoid space extension (Figure 4E).

In the fourth ventricle, the CP is attached both to the inferior part of the cerebellum (Figure 5A, arrowheads) and the superior part of the brain stem. While the bulk of the CP is localized close to the cerebellum, the connection of the CP toward the brain stem is mediated *via* a delicate tissue bridge (Figures 5B–D, tissue bridge marked by arrowhead in Figure 5C). At the median cutting plane, this tissue bridge connects to the area postrema of the hindbrain (Figure 5C). While directly lateral to this point, the CP is continuously connected to the brainstem via this inferior connection (Figure 5D), at about 400 μm lateral from the median plane, this inferior connection detaches from the brainstem, thereby forming the opening between the fourth ventricle and the subarachnoid space (Figure 5E).

During the evaluation of the CP morphology, the close spatial relationship of the CP attachment regions and the subarachnoid space appeared notably remarkable to us. In the fourth ventricle, the CP is spanned between the cerebellum and brainstem, forming the dividing structure between the fourth ventricle facing the CP epithelium and the subarachnoid space facing the CP stroma (Figure 5). In adjacency to the third ventricle, the spatial structure of the subarachnoid space shows a higher complexity. As introduced in Figure 4, the subarachnoid space extends at the roof of the third ventricle between the brain parenchyma and the CP. The spatial reconstruction showing the connection of this space to the subarachnoid space of the brain surface is shown in Figures 6A,B. In addition, we used sagittal histologic sections for immunohistochemical labeling of laminin to identify brain parenchyma surfaces facing the subarachnoid space by the presence of a basal lamina. In the mediosagittal cutting plane, the connection of the subarachnoid space and the ventricular system is most prominent. The subarachnoid space extends from the outer brain surface between the posterior part of the retrosplenial cortex and the superior colliculus of the midbrain to reach the third ventricle’s roof (Figure 6C, arrowheads). It then protrudes anteriorly between the corpus callosum and the CP of the third ventricle toward the posterior aspect of the subfornical organ to form an elongated cavity of subarachnoid space above the CP (Figures 4E, 6E,F). Further laterally, the subarachnoid space of the brain surface establishes a connection to the lower aspect of the lateral ventricle. This connection runs between the diencephalon and the retrosplenial cortex, hippocampal formation, and the hippocampal fimbria (Figure 6D, arrowheads). In total, the subarachnoid space forms a downward-curved plane between the posterior telencephalon and the diencephalon following the shape of the lateral ventricles (Figures 6A,B). The thickness of this subarachnoid space is variable depending on the location. In the median aspect, it forms a widened space (Figures 6C,E,F), while further lateral, the upper telencephalic and lower diencephalic brain surfaces are in direct contact with each other (Figure 6D). Only single channels, often filled by meningeal blood vessels running in the subarachnoid space, appear opened.

## Discussion

We here present a micro-CT-based description and three-dimensional reconstruction of the murine ventricle system and CP supplemented by histological and immunohistochemical data. Micro-CT scanning of fixated and immersion-contrasted murine brains resulted in high-resolution image stacks of the complete murine brain. In our experience, these image stacks proved helpful for the planning of histological sectioning of specific locations and orientation in histologic sections in addition or even as an alternative to histologic atlases that are often incomplete for sagittal and horizontal sectioning planes.

Alignment of micro-CT datasets and histological sections resulted in high congruence in all cutting planes. However, some morphological spaces appeared to be dilated to different extents in the two methods. This could be due to different tissue shrinking during sample preparation. For both methods, murine brains are fixated by transcardial perfusion with formaldehyde solution. Afterward, brains for histologic sectioning were embedded in paraffin, and brains for micro-CT scanning were immersion-contrasted in Lugol’s iodine for two weeks. Fixation by formaldehyde is known to result in considerable tissue shrinkage (Anderson and Maga, 2015), as does embedding in paraffin (Quester and Schröder, 1997) and prolonged immersion in Lugol’s iodine (Dawood et al., 2021). Therefore, an influence on the dimensions of morphological structures or cavities in the murine brain is highly probable.

In our descriptions of the murine CP, we focused on its attachment sites toward the brain parenchyma because these regions might play a role in immune cell migration of peripheral immune cells into the CNS under inflammatory conditions. The fenestrated endothelium of blood vessels without specific barrier properties in the CP stroma enables facilitated egress of circulating immune cells into the CP stroma. In progressive multiple sclerosis, for instance, elevated immune cell densities in the CP stroma have been demonstrated in post-mortem material (Rodríguez-Lorenzo et al., 2020). From the CP stroma, activated immune cells can breach the blood-CSF barrier on the level of the CP epithelium to enter the CSF. In addition, Llovera et al. (2017) observed immune cell recruitment in a mouse model of ischemic stroke from the CP into the CNS after blockage of the ventricular lumen by matrigel injection. They describe the attachment of the CP to the lateral ventricle wall near the interventricular foramen and its close relationship to blood vessels of the subarachnoid space. Additionally, they were able to locate T cells along the proposed migration route following the corpus callosum to the peri-infarct cortex. To our knowledge, there is no further evidence for the migration of peripheral immune cell migration along the attachment region of the CP available. However, the close spatial relationship of the CP stroma and the CNS parenchyma in all four ventricles is a strong argument for the relevance of the attachment region in immune cell recruitment.

Our results further indicate that the subarachnoid space of the brain surface has a direct connection to all four ventricles in mice. A connection between the subarachnoid space and the lateral ventricle has already been described in the rat in studies of tracer-dependent CSF distribution. It is under discussion whether the connection between the subarachnoid space and the ventricles might serve as an alternative route for CSF flow in case of obstruction of the mesencephalic aqueduct (Ghersi-Egea et al., 1996; Park et al., 2011; Yoon et al., 2015; Bedussi et al., 2017).

Imaging of the ventricle system and the CP is a standard diagnostic procedure in the clinical investigation of many neurological diseases, and a growing number of CP-related biomarkers is proposed as diagnostic or prognostic markers for diseases like schizophrenia, epilepsy, and Alzheimer’s disease (reviewed in Hubert et al., 2019). In research on murine models, CP imaging is rarely applied. However, Fleischer et al. (2021) recently demonstrated that the CP volume, reconstructed from magnetic resonance imaging (MRI), is elevated in multiple sclerosis patients and two mouse models of multiple sclerosis. The mechanisms behind CP enlargement remain unresolved to date, but these results indicate that imaging of the CP in mice might be a valuable additional read-out in experimental mouse models of neurologic diseases.

In conclusion, we here provide highly detailed micro-CT data sets of the murine brain with three-dimensional reconstructions of the ventricles and the CP as a reference for future studies on this system. The detailed description of the attachment of the CP toward the CNS parenchyma and its close spatial relationship to the subarachnoid space will be the basis for further analysis of a potential role in immune cell migration under neuroinflammatory conditions.

## Data availability statement

The raw data supporting the conclusions of this article will be made available by the authors, without undue reservation.

## Ethics statement

The animal study was reviewed and approved by Landesamt für Landwirtschaft, Lebensmittelsicherheit und Fischerei Mecklenburg-Vorpommern.

## Author contributions

SJ and MK provided study conception and design. TG, KM, LB, and HK performed transcardial perfusion, tissue preparation for histology, histological stainings, and immunohistochemical labeling. JR prepared and conducted the micro-CT scans. JR and JK did the 3D-reconstruction and visualization of micro-CT data. TG and SJ prepared the figures and schematic images. SJ wrote the manuscript. All authors reviewed the results and manuscript and approved the final version of the manuscript.

## Supplementary material

Supplementary Figure 1 Variation in size of morphological spaces and fissures. The width of fissures within the brain varied in size between animals and occasionally between hemispheres within the same animal. **(A–A”)** Sectional planes from micro-computed tomography scans and **(B–B”)** hematoxylin-eosin staining of frontal sections at the level of the rostral hippocampus from three animals per imaging method. Large openings of the space between hippocampal formation and diencephalon are marked by arrowheads. Scale bar 300 μm.

Supplementary Figure 2 Attachment of the choroid plexus in the lateral ventricle. **(A)** Immunofluorescence double labeling with anti-laminin (green), anti-aquaporin1 (AQP1, red), and nuclear staining with 4′,6-diamidino-2-phenylindole (DAPI, blue) and **(B)** hematoxylin-eosin staining of the attachment region of the choroid plexus in the lateral ventricle in a section with artificially widened subarachnoid space between the hippocampal fimbria and the diencephalon. Two tissue bridges (arrowheads) with separate continuous basal laminae form the attachment of the choroid plexus. fi hippocampal fimbria, LV lateral ventricle, SAS subarachnoid space.
