# Supplementary figures and images for: Morphology of the murine choroid plexus: Attachment regions and spatial relation to the subarachnoid space
Source: Front Neuroanat. 2022 Oct 31;16:1046017. doi: 10.3389/fnana.2022.1046017 (PMC9659632; doi:10.3389/fnana.2022.1046017)

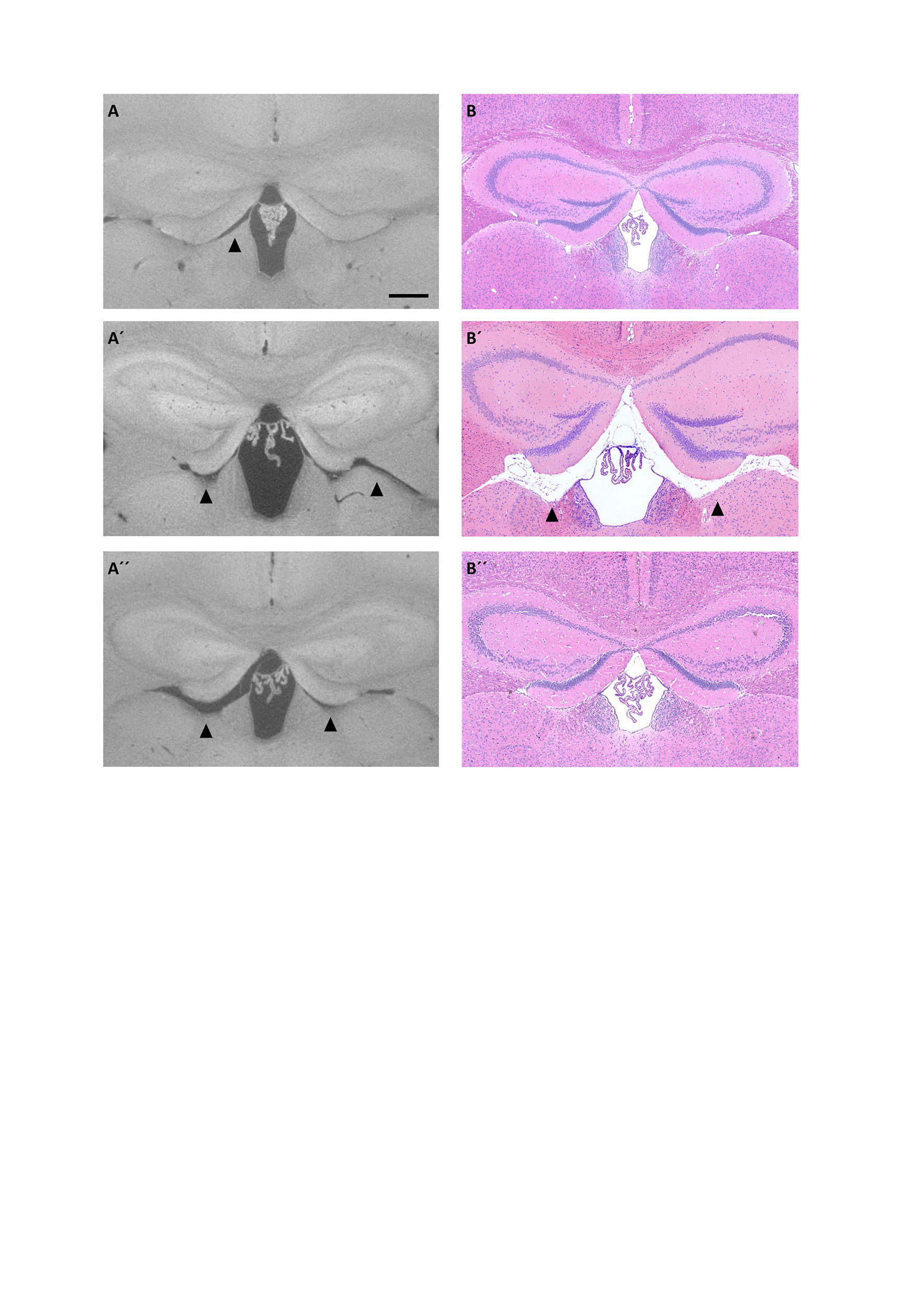

Supplement: Supplementary Figure 1 — Variation in size of morphological spaces and fissures. The width of fissures within the brain varied in size between animals and occasionally between hemispheres within the same animal. (A–A”) Sectional planes from micro-computed tomography scans and (B–B”) hematoxylin-eosin staining of frontal sections at the level of the rostral hippocampus from three animals per imaging method. Large openings of the space between hippocampal formation and diencephalon are marked by arrowheads. Scale bar 300 μm. [file Image_1.JPEG]

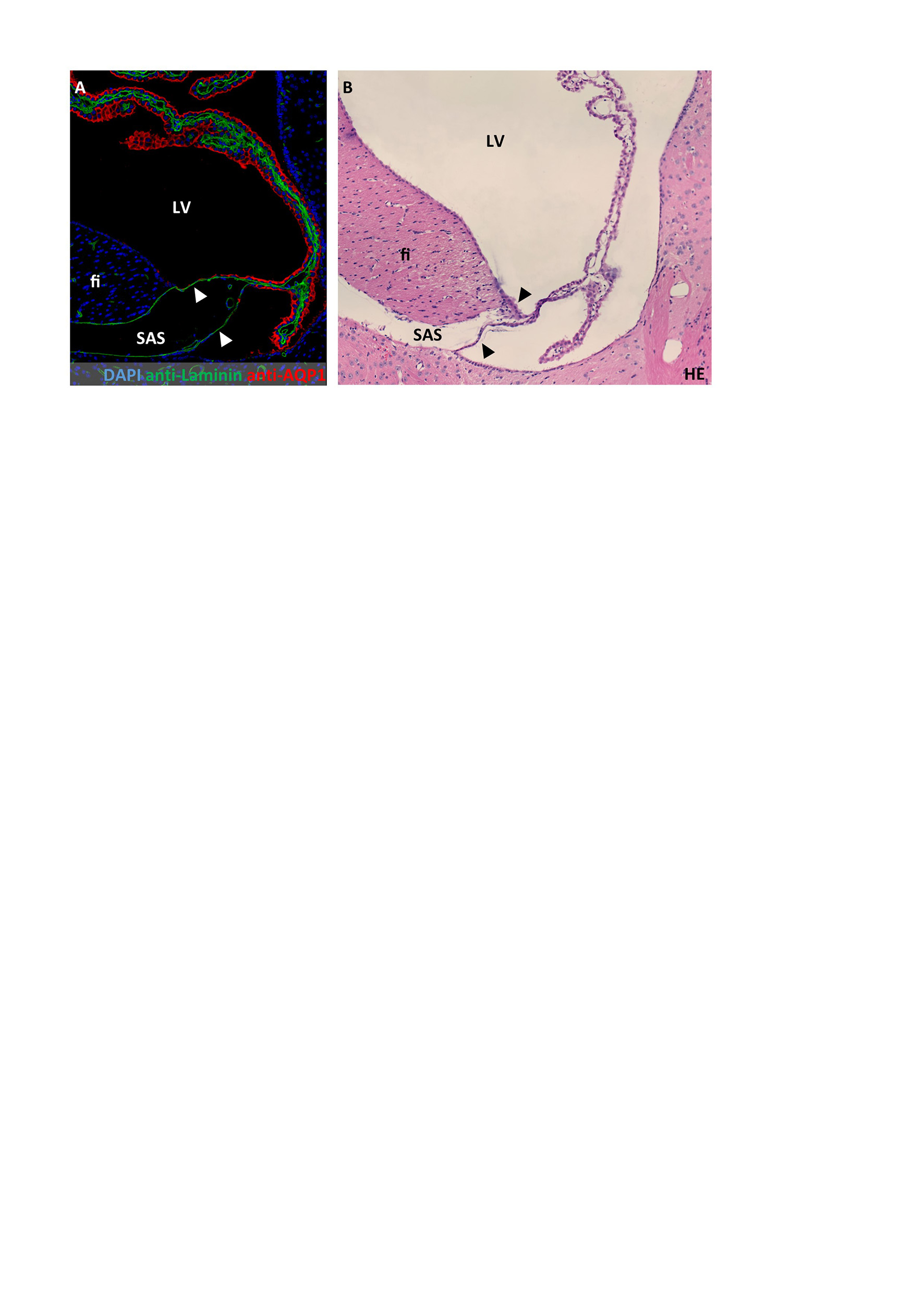

Supplement: Supplementary Figure 2 — Attachment of the choroid plexus in the lateral ventricle. (A) Immunofluorescence double labeling with anti-laminin (green), anti-aquaporin1 (AQP1, red), and nuclear staining with 4′,6-diamidino-2-phenylindole (DAPI, blue) and (B) hematoxylin-eosin staining of the attachment region of the choroid plexus in the lateral ventricle in a section with artificially widened subarachnoid space between the hippocampal fimbria and the diencephalon. Two tissue bridges (arrowheads) with separate continuous basal laminae form the attachment of the choroid plexus. fi hippocampal fimbria, LV lateral ventricle, SAS subarachnoid space. [file Image_2.JPEG]
