# Supplementary material for: Morphology of the murine choroid plexus: Attachment regions and spatial relation to the subarachnoid space
Source: Front Neuroanat. 2022 Oct 31;16:1046017. doi: 10.3389/fnana.2022.1046017 (PMC9659632; doi:10.3389/fnana.2022.1046017)

Morphology of the murine choroid plexus: attachment regions and spatial relation to the subarachnoid space

Theresa Greiner, Katerina Manzhula, Louise Baumann, Hannes Kaddatz, Jens Runge, Jonas Keiler, Markus Kipp, Sarah Joost

Supplementary Material 1

Three-dimensional surface model of the ventricle, choroid plexus, and subarachnoid space

This pdf contains an interactive three-dimensional surface model of the brain parenchyma (yellow), ventricle system (blue), the choroid plexus (purple), and the subarachnoid space (red) that is in contact with the lateral ventricle based on micro-CT. The different aspects of the model can be switched on or off after activating three-dimensional properties in pdf readers that support three-dimensional objects.

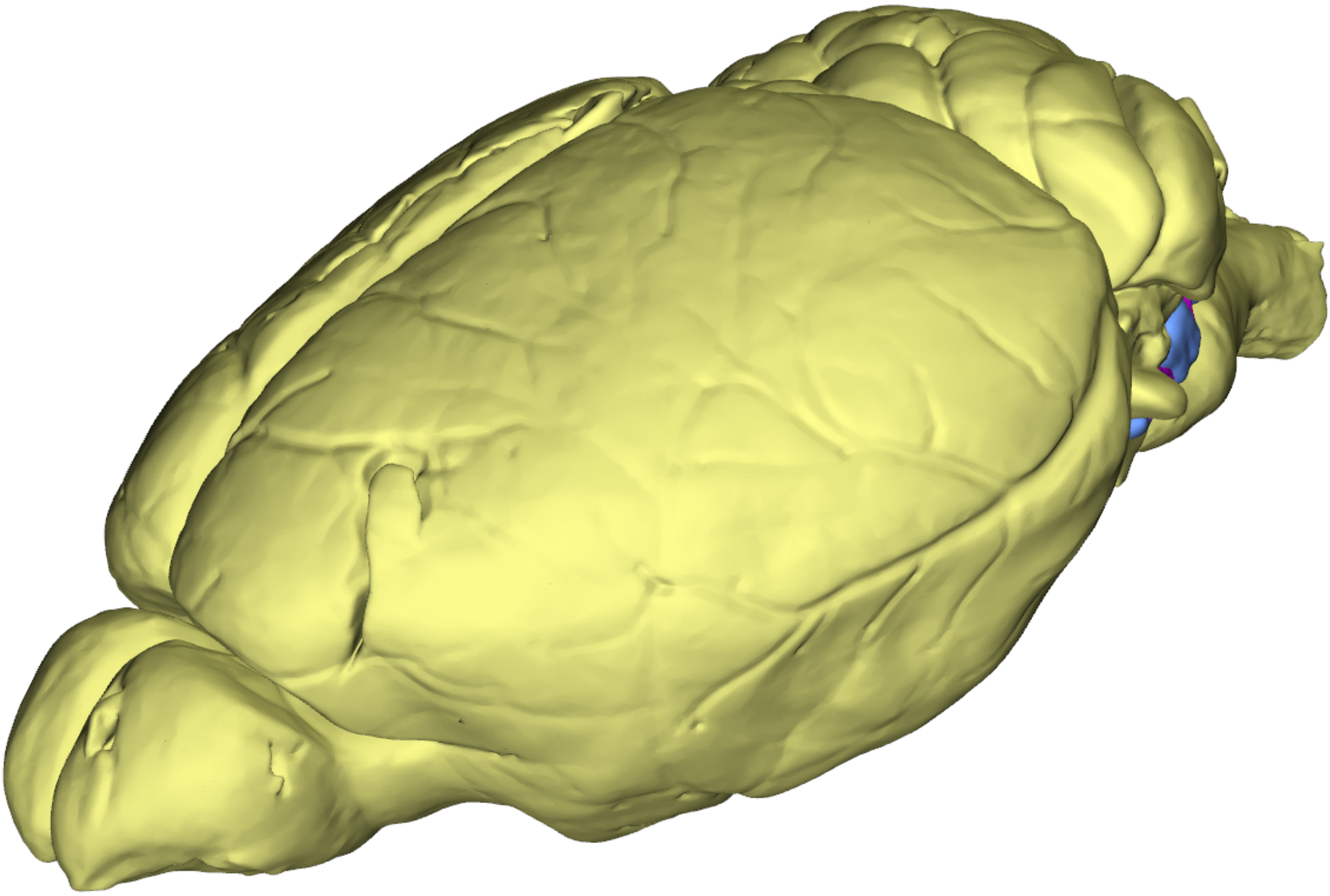

Supplement: Supplementary file 3 [file Data_Sheet_1.pdf]
